# Healthy Donors Harbor Memory T Cell Responses to RAS Neo-Antigens

**DOI:** 10.3390/cancers12103045

**Published:** 2020-10-19

**Authors:** Morten Orebo Holmström, Mads Hald Andersen

**Affiliations:** 1 National Center for Cancer Immune Therapy, Department of Oncology, Herlev Hospital, DK-2730 Herlev, Denmark; mads.hald.andersen@regionh.dk; 2 Institute for Immunology and Microbiology, Copenhagen University, DK-2200 Copenhagen, Denmark

**Keywords:** RAS, immuno-editing, immune surveillance, T cell memory, neo-antigens

## Abstract

**Simple Summary:**

Memory T cells from healthy individuals respond to stimulation with peptides derived from cancer-specific *RAS* mutations, thus suggesting that healthy individuals have been challenged with *RAS* mutations earlier in life.

**Abstract:**

The *RAS* mutations are the most frequently occurring somatic mutations in humans, and several studies have established that T cells from patients with *RAS*-mutant cancer recognize and kill *RAS*-mutant cells. Enhancing the T cell response via therapeutic cancer vaccination against mutant *RAS* results in a clinical benefit to patients; thus, T cells specific to *RAS* mutations are effective at battling cancer. As the theory of cancer immuno-editing indicates that healthy donors may clear malignantly transformed cells via immune-mediated killing, and since T cells have been shown to recognize *RAS*-mutant cancer cells, we investigated whether healthy donors harbor T-cell responses specific to mutant RAS. We identified strong and frequent responses against several epitopes derived from the RAS codon 12 and codon 13 mutations. Some healthy donors demonstrated a response to several mutant epitopes, and some, but not all, exhibited cross-reactivity to the wild-type RAS epitope. In addition, several T cell responses were identified against mutant *RAS* epitopes in healthy donors directly ex vivo. Clones against mutant RAS epitopes were established from healthy donors, and several of these clones did not cross-react with the wild-type epitope. Finally, CD45RO^+^ memory T cells from healthy donors demonstrated a strong response to several mutant RAS epitopes. Taken together, these data suggest that the immune system in healthy donors spontaneously clears malignantly transformed *RAS*-mutant cells, and the immune system consequently generates T-cell memory against the mutations.

## 1. Introduction

The theory of cancer immuno-editing stipulates that transformed cells can be recognized and killed by host immune cells before the development of cancer [1,2]. Although generally accepted, the theory has been difficult to prove in humans. However, some studies have indicated that T cell-mediated recognition of transformed cells may protect against the development of cancer. Individuals with human leukocyte antigen (HLA)-A3 and HLA-B8 are underrepresented in *BCR-ABL*^+^ chronic myeloid leukemia (CML) [3], and individuals with HLA-B*07, B*18, or B*40 have a lower risk of nucleophosmine-1 (*NPM1*)-mutant acute myeloid leukemia (AML) [4]. This suggests that neo-antigens encoded by *NPM1* mutations or the *BCR-ABL* fusion gene are presented effectively by certain HLA types, facilitating T cell-mediated killing of the transformed cells. T cell-mediated antigen recognition of transformed cells generates tumor antigen-specific memory T cells, which can be analyzed in cell studies and used in cancer immune therapy [5]. Studies have shown that healthy donor (HD) T cells recognize antigens encoded by somatic *NPM1* mutations [6], and the tumor-associated antigen melanoma antigen recognized by T cells-1 (MART1) [7]. However, MART1-specific T cells exhibit a naïve phenotype [8], and the phenotype of NPM1-specific T cells has not been investigated. Recently, we showed that the calreticulin (*CALR*) exon 9 mutations, which are identified in 20% of patients with chronic myeloproliferative neoplasms (MPN) [9,10] encode highly immunogenic neo-antigens [11,12]. In addition, we have shown that HDs have memory T cell responses to CALR-mutant neo-antigens, and these responses are more frequent and stronger than the responses identified in patients [13]. Moreover, sorted memory T cells from HDs are specific to mutant CALR antigens [13], which suggests that the immune system in HDs has cleared *CALR* exon 9 mutant cells, providing evidence of tumor immune surveillance against these mutations.

RAS proteins are proto-oncogenes encoded by *HRAS*, *KRAS,* and *NRAS*, and are the most frequently mutated genes in cancer [14]. They are GTPases that work as molecular switches to regulate pathways implicated in cell survival and proliferation [14]. The majority of RAS mutations are single amino acid substitutions in codon 12, 13, or 61, and mutations at these sites induce GTP binding and constitutive activation of RAS [14]. All *RAS* genes have sequence homology at these mutational sites. Given the high frequency of these mutations in cancer, several trials have investigated the occurrence of T cell responses to antigens encoded by *RAS* mutations [15,16,17,18]. Some studies have shown that patients with *RAS*-mutant cancer harbor T cells specific to the *RAS* mutations [15,16,17]. These findings have spurred several clinical trials with therapeutic cancer vaccines aiming to enhance the T-cell responses to *RAS* mutations [19,20,21,22]. Concurrently, some studies have investigated but failed to identify *RAS* mutation-specific T cells in HDs, although several reports have shown that it is possible to induce *RAS* mutation-specific T cells in HDs and patient peripheral blood mononuclear cells (PBMCs) after repeated peptide stimulation [23,24,25,26,27]. However, primary responses specific to RAS mutant neo-antigens have so far not been identified in T cells from HDs [16,17,18,25].

Given that we were able to detect T cell responses specific to *CALR* exon 9 mutations, and that *RAS* mutations are the most frequent mutations in cancer, we sat out to investigate if HDs harbor T cells specific to RAS mutation-derived neo-antigens. We show that a high proportion of HDs harbor T cells that are specific to neo-antigens derived from codon 12 and 13, but not codon 61, mutations. We generated T cell clones specific to several of the codon 12 mutations and show that the majority of these clones do not cross-react with the wild-type epitope of RAS. Finally, we show that the *RAS* mutation-specific T cells in HDs exhibit a memory phenotype, which suggests the existence of tumor immune surveillance against the most frequent somatic mutation in human cancer.

## 2. Results

### 2.1. Initial Screening Against RAS-Mutant Crude Epitopes Reveal Strong and Frequent Responses to Several RAS-Mutant Neo-Antigens

**Figure 1:**
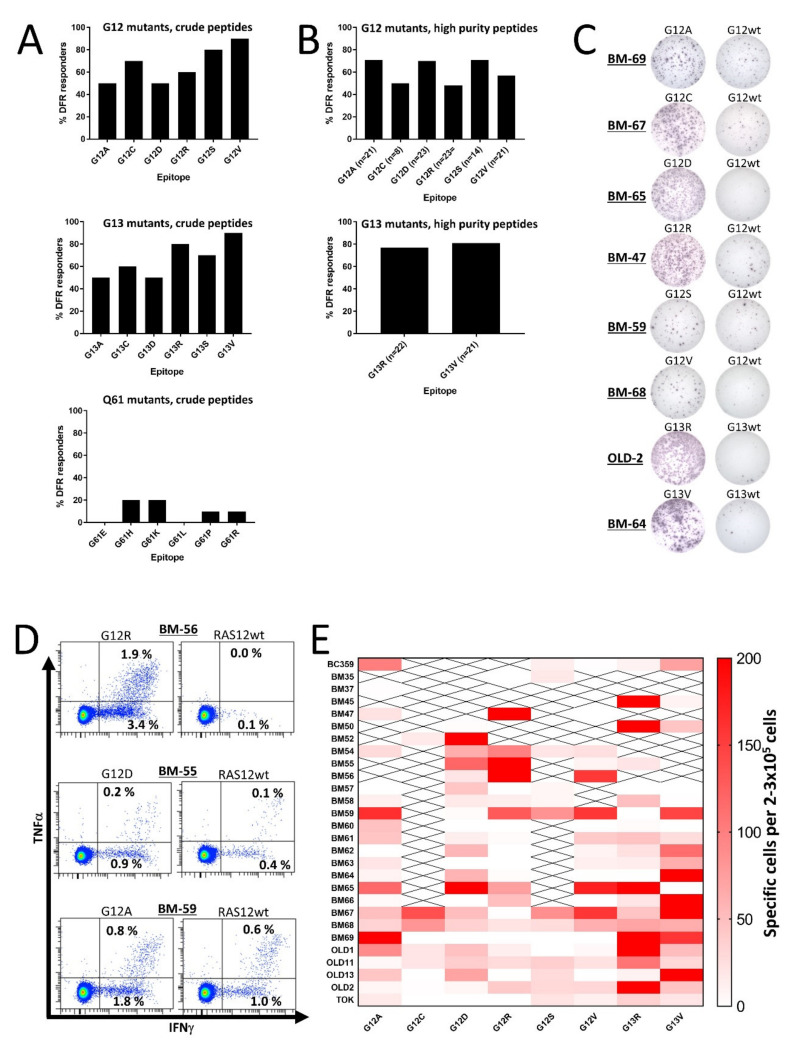
In vitro interferon (IFN)-γ enzyme-linked immunospot (ELISPOT) responses against RAS-mutant neo-antigens in healthy donor peripheral blood mononuclear cells (PBMCs). PBMCs were stimulated once in vitro and the next day with low-dose interleukin (IL)-2, and then incubated for 12–14 days before the restimulation of cells in a IFN-γ ELISPOT. (**A**) The frequency of responses in PBMCs from 10 healthy donors (HDs) against crude RAS mutant peptide epitopes derived from the codon 12 (top), codon 13 (middle), and codon 61 (bottom) mutations. Responses were defined according to the distribution-free resampling (DFR) method [28]. (**B**) The frequency of responses against high-purity RAS mutant peptide epitopes derived from the codon 12 mutations (top), and those against the G13R and G13V substitutions derived from the codon 13 mutations (middle). Responses were defined according to the distribution-free resampling (DFR) method [28]. The *n* = *xx* depicted under each bar shows how many donors were tested against the peptide of interest. (**C**) Representative raw results from panel B. (**D**). Examples of responses identified by intracellular cytokine staining in healthy donor CD4^+^ T cells against the G12R, G12D, and G12A peptides. (**E**) Heatmap depicting the amplitude of the response against the high-purity peptide epitopes, with the wild-type (wt) epitope as a negative control. To reach the normalized count per donor, the mean number of spots in the neo-antigen-stimulated wells was subtracted by the mean count in the wt-stimulated wells. All experiments were performed with 2–3 × 10^5^ cells/well. “X” signifies that the appropriate donor has not been tested against the epitope.

Initially, we scrutinized HD T cells for responses to mutant RAS epitopes. To expand the number of 9-mer CD8 epitopes for each peptide, we chose to work with 19-mer peptide epitopes derived from codon 12, 13, or 61 mutations with the single amino acid substitution at the central position. For codons 12 and 13, the most frequent substitutions are G to A, C, D, R, S, or V, and for codon 61 they are Q to E, H, K, L, P, or R [14]. Therefore, we generated six mutant epitopes for each codon with a purity >70%, resulting in a total of 18 peptides for our analyses (Table 1). PBMCs from 10 HDs were analyzed for spontaneous immune responses against the RAS-mutant epitopes using interferon (IFN)-γ enzyme-linked immunospot (ELISPOT) assays, as described previously [11]. Keeping the negative HD T cell responses from previous studies in mind, we did not expect to find any strong responses against the epitopes; as such, we did not include the corresponding wild-type (wt) epitope as a negative control. However, we were surprised to find that both codon 12 and codon 13 mutant epitopes incited strong release of IFN-γ from stimulated HD PBMCs (Figure S1), as at least 50% of the HDs presented a significant response, according to the distribution-free resampling (DFR) rule [28] against each of the six epitopes derived from the codon 12 and codon 13 mutations (Figure 1A). The codon 12 epitopes were uniformly immunogenic but of the codon 13 epitopes, the R and V substitutions were more immunogenic than the other epitopes. The findings regarding codon 61 mutations were more on par with previous findings, as we only identified some weak responses to peptides with the H, K, P, and R substitutions (Figure 1A and Figure S1).

### 2.2. T Cell Responses to Mutant RAS Protein Are Not Explained by Either Peptide Impurities or Cross-Reactivity with the Wild-Type Epitope

The strong and frequent responses described above led us speculate whether they can be explained by cross-reactivity with the wt epitope of RAS. Thus, we performed IFN-γ ELISPOT assays using peptides with a purity >90%; most importantly, cells were stimulated with the corresponding wt epitopes as negative controls in the ELISPOT wells. We chose to analyze responses to all epitopes from the codon 12 mutations, because they are the most frequent *RAS* mutations [14,29]. We also included high-purity epitopes from the G13R and G13V mutations, as they were the most immunogenic peptides in our initial analyses. As the codon 61 mutations did not demonstrate any immunogenic potential, we chose not to continue our work with the codon 61 mutations. T cells with cross-reactivity to the wt epitope were thought to explain the high frequency of RAS-mutant-specific immune responses; however, we found that HD T cells still demonstrated RAS-mutant-specific immune responses, even when the negative controls were PBMCs stimulated with wt peptides (Figure 1B,C and Figure S2). The extent of the responses remained high but were slightly different from the responses against the crude epitopes. The responses between the crude and high-purity peptides were not evenly distributed as more HDs demonstrated a response against the high-purity G12A and G12D peptides, whereas fewer responses were identified against G12C, G12R, G12S, and G12V peptides (Figure 1B). Surprisingly, the responses against the G13R and G13V high-purity epitopes remained high (Figure 1B). The responses in experiments with wt peptide-stimulated negative controls differed from experiments with unstimulated negative controls. Hence, in order to investigate any difference in response to the mutant epitopes we performed experiments in 18 HDs with both controls and found no significant difference between experiments in which unstimulated cells were used as negative controls and experiments in which wt peptide-stimulated cells were used as negative controls (Figure S3). Using intracellular cytokine staining (ICS), we investigated the response against G12A in two donors, against G12D in two donors, and against G12R in four donors. A CD4^+^ T cell response was identified in all donors against G12A and G12D, and three of four donors displayed a CD4^+^ T cell response against G12R (Figure 1D). In these experiments, some cross-reactivity to the wt epitope was demonstrated; however, cultures displayed a markedly higher release of IFN-γ and tumor necrosis factor (TNF)-α upon stimulation with a mutant epitope compared to stimulation with a wt epitope. No CD8^+^ T cell responses were identified in any of the donors (Figure S4).

### 2.3. T Cells from Healthy Donors Demonstrate a Strong Response to Stimulation with Several Different Mutant RAS Epitopes

Previous studies have shown that T cells from patients immunized with mutant RAS epitopes react to stimulation with several mutant RAS epitopes, and some patient T cells cross-react with the wt epitopes [15,30]. We also found that the majority of tested donors harbored T cells that responded to several mutant RAS epitopes. Some donors even demonstrated strong responses to several of the epitopes (Figure 1E). Interestingly, a few donors had an inverse response, in which the strongest immune responses were detected against the wt epitope. Donor BM69 and OLD13 in particular presented strong responses against the wt epitope, but the majority of the donors did not exhibit any response to the wt epitope.

### 2.4. T Cells from Healthy Donors Respond to Stimulation with Mutant RAS Epitopes in an Ex Vivo Setting

**Figure 2:**
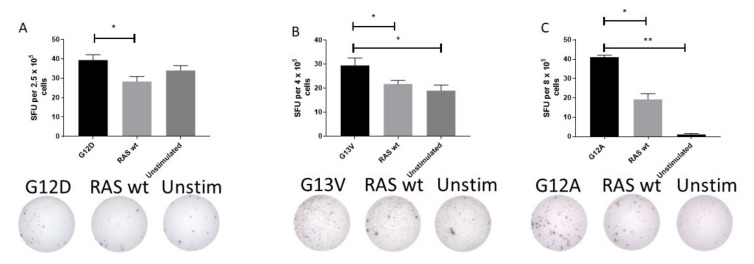
Ex vivo immune responses against mutant epitopes in three different healthy donors, with the wild-type (wt) epitope and unstimulated cells as negative controls. Healthy donor peripheral blood mononuclear cells were thawed and rested over night before they were plated directly ex vivo into the ELISPOT plate and stimulated with the appropriate peptides for 48 h before development of the plate. The experiments included both wt peptide-stimulated cells and unstimulated cells as negative controls. Response to G12D (**A**), G13V (**B**), and G12A (**C**) epitopes are shown. * indicates a DFR-defined response, whereas ** indicates a DFR(2x) defined response [28].

The surprisingly high frequency of T cell responses specific to mutant epitopes led us to investigate whether HD T cells exhibit an immune response to mutant RAS epitopes in an ex vivo setting. Therefore, PBMC from HDs with a strong immune response to mutant RAS epitopes were stimulated with peptides for 24 or 48 h ex vivo without any prior in vitro stimulation. Fourteen donors were tested in ex vivo ELISPOT assays against one or several peptides, with a median number of tested peptides of 1 per donor (range 1–3). Donor BM-52 was tested twice against G12D. In one experiment, we identified a DFR-defined immune response with the RAS wt epitope as a negative control (Figure 2A), and in another experiment we identified a DFR-defined immune response with unstimulated wells as negative controls (data not shown). Donor BM-64 was tested once against the G13V epitope in a TNF-α ELISPOT, and demonstrated a DFR-defined immune response with both the RAS wt peptide and unstimulated wells as controls (Figure 2B). Donor BM-60 was tested twice against the G12A peptide, and in one experiment we found a DFR-defined immune response against the unstimulated negative control, but not RAS wt (data not shown). However, upon repetition of the experiment, we identified a DFR(2x) defined immune response with unstimulated wells as negative controls, and a DFR-defined immune response with RAS wt as the negative control (Figure 2C). Interestingly, G12D mutation is the most common mutation in pancreatic adenocarcinoma, with approximately 90% of patients harboring a mutation in *KRAS* [31].

### 2.5. T-Cell Clones Reactive to Mutant RAS Epitopes May Express Some Cross-Reactivity to Wt RAS Epitopes

**Figure 3:**
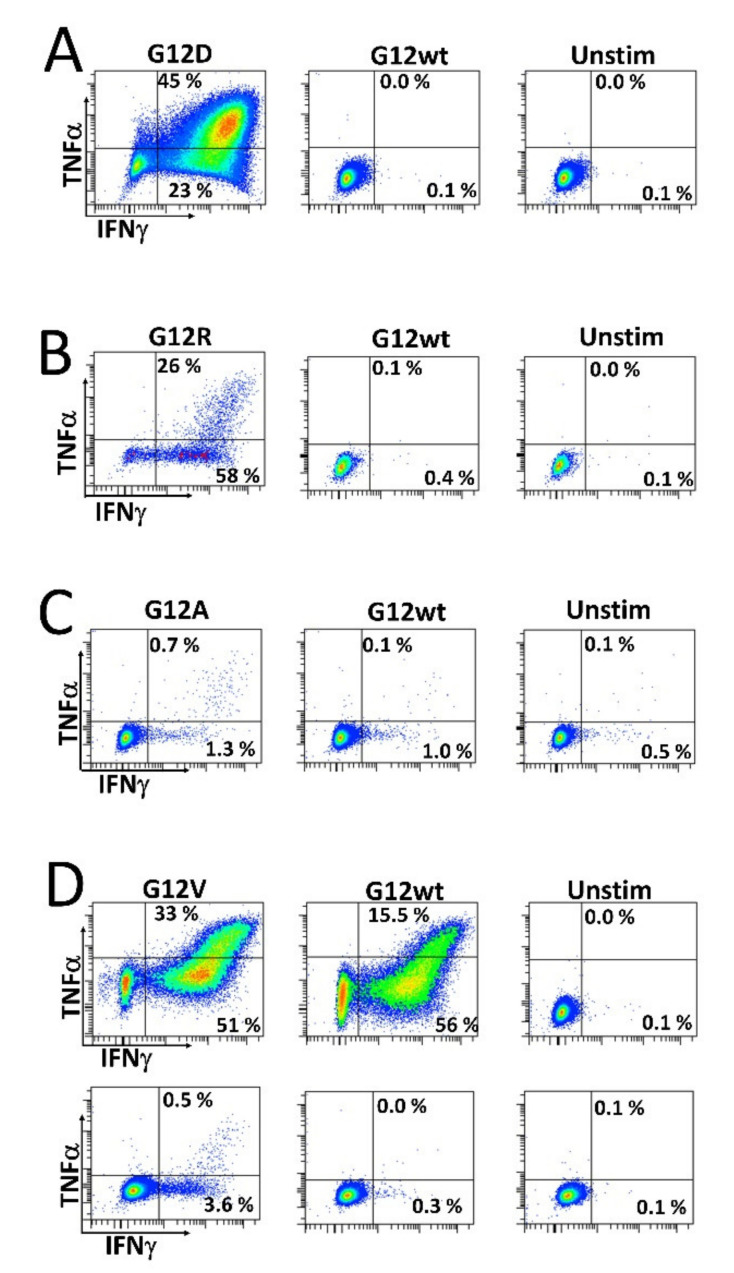
Stimulation of CD4^+^ T cell cultures established from healthy donor peripheral blood mononuclear cells. T cell clones were established as described in the Materials and Methods section and were subsequently stimulated with their corresponding mutant RAS epitope, wt RAS epitope, or left unstimulated. (**A**) Response against the G12D epitope and (**B**) G12R epitope response in a CD4^+^ T cell culture. (**C**) Response against the G12A epitope in a CD4^+^ T cell culture established by ex vivo stimulation as described the Materials and Methods section. (**D**) Response specific to the G12V peptide in a CD4^+^ T cell culture, with the standard 5 µM concentration of peptide (top) and 0.5 µM concentration (bottom).

As we wanted to investigate whether HD T cells that are reactive to mutant RAS epitopes potentially cross-react with the wt epitope, we established specific T cell cultures by stimulating HD PBMCs in vitro with mutant RAS peptide, and then with IL-2 the next day, before the cells were expanded in culture for 14 days. The cells were re-stimulated with peptide for 18 h before enriching specific cells by magnetically activated cell sorting (MACS) selecting for CD137^+^ (also known as 4.1 BB) cells. Cells were then cloned by limiting dilution with 1 cell/well and expanded using our rapid expansion protocol (REP). Cells were tested for an antigen response when an appropriate number of cells had expanded. As such, we established highly specific T cell cultures against both the G12R and G12D mutant epitopes. Interestingly, these highly specific cultures did not exhibit cross-reactivity with the wt epitope (Figure 3A,B). Next, we established a G12A-specific T cell culture by enriching *ex vivo*-stimulated cells. Briefly, HD PBMCs were thawed, rested overnight, re-stimulated the next day with peptide, and allowed to incubate for 18 h before enriching CD137^+^ cells using MACS. The enriched cells were then expanded using the REP. Analysis of the enriched and expanded T cells revealed a response to both the mutant G12A epitope and wt epitope, but with a stronger response to the G12A mutant epitope (Figure 3C). We also established a T cell culture specific to the G12V epitope using two in vitro stimulations, as described above. This T cell line showed considerable cross-reactivity with the wt epitope at a peptide concentration of 5 µM (Figure 3D, top). At a concentration of 0.5 µM, the response to the wt epitope was abrogated (Figure 3D, bottom), showing that the T cell receptor of the specific T cells has a higher avidity for the mutant epitope than the wt epitope.

### 2.6. RAS-Mutant-Specific T Cells in Healthy Donors Are Antigen-Experienced Memory T Cells

The strong responses against the mutant RAS epitopes identified above suggest that the T cells specific to mutant RAS are memory T cells, as these cells more readily secrete IFN-γ upon antigen stimulation compared to naïve T cells [32]. To investigate this, we enriched CD4^+^CD45RO^+^ memory T cells using MACS, and stimulated the enriched cells once in vitro, allowing the T cells to expand, then re-stimulated the T cells and analyzed them for a response against mutant RAS in IFN-γ ELISPOT assays. However, we only detected very weak and non-convincing responses using this method (data not shown). We speculated that the absence of a response could be due to the low capacity of CD4^+^CD45RO^+^ memory cells to process and present antigens, as our enrichment method only included CD4^+^CD45RO^+^ memory cells in the in vitro culture.

**Figure 4:**
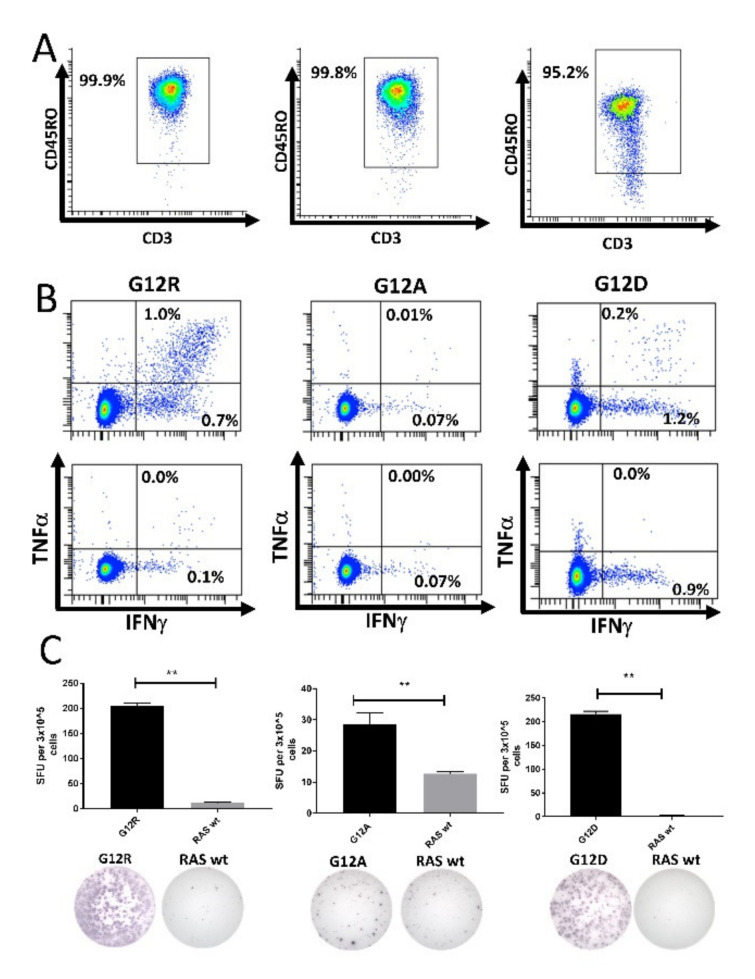
High-purity cultures of CD4^+^CD45RO^+^ memory T cells respond to stimulation with mutant RAS epitopes without cross-reactivity to the wild-type (wt) epitope. Left: Healthy donor BM-56; middle: Healthy donor BM-59; right: Healthy donor BM-65. (**A**) Purity analysis of the enriched CD4^+^CD45RO^+^ memory T cells mixed with peptide-pulsed, autologous CD14^+^ monocytes. The gate was set for live CD3^+^ CD4^+^ cells to investigate the purity of the enriched cells. (**B**) Fluorescence-activated cell sorting (FACS) plots from an intracellular cytokine stain in which enriched memory cells were stimulated with mutant epitope (top) or wt epitope (bottom). (**C**) IFN-γ ELISPOT assays with a graphical depiction of the response (top) and representative wells from the experiment (bottom). ** indicates a DFR(2x) defined response [28].

Next, we isolated CD14^+^ monocytes using MACS. These cells were pulsed with peptide for 2 h before being used to stimulate isolated CD4^+^CD45RO^+^ memory T cells. The purity of the isolated memory T cells was assessed by fluorescence-activated cell sorting (FACS) (Figure 4A and Figure S5). The memory cells were incubated for 12 days in culture before analysis by ELISPOT and ICS. We enriched CD4^+^ memory T cells from three different HDs that had earlier displayed a strong response to the G12A, G12D, or G12R epitopes. The enriched memory cells exhibited strong responses to the mutant RAS epitopes in both ICS (Figure 4B) and IFN-γ ELISPOT assays (Figure 4C). No response was identified against the wt RAS epitopes in the isolated T cells. As we had sufficient cryopreserved cells from the donor with a response to RAS G12R, we performed the experiment again, and once more showed that HD T cells specific to mutant RAS antigens are antigen-experienced memory T cells (Figure S6).

## 3. Discussion

In the current study, we describe that HDs present strong and frequent in vitro T cell responses to epitopes derived from the *RAS* codon 12 and codon 13 mutations. Cross-reactivity to the corresponding wt epitope was only identified in some donors, and PBMCs plated directly ex vivo presented a response to mutant epitopes. T cell cultures with high specificity to mutant RAS epitopes exhibited low to no cross-reactivity with the wt epitope. Importantly, HD T cells that responded to stimulation with mutant RAS peptides exhibited an antigen-experienced memory T cell phenotype. Taken together, these data suggest that HDs harbor an intact *RAS*-mutant-specific immune surveillance that could protect against the occurrence of *RAS*-mutant cancer.

The *RAS* mutations are an intriguing target for the treatment of cancer. Starting in the early 1990s, several studies investigated the occurrence of T cell responses specific to the *RAS* mutations in HDs and patients with *RAS*-mutant cancer [17,18,25]. In order to identify whether mutant cells could be targeted by specific T cells for cancer immune therapy, several studies have demonstrated that patients with *RAS*-mutant cancer harbor T cells specific to mutant RAS epitopes [16,17]. Previously, no studies have identified primary responses in HDs [16,17,18,25], but several studies have demonstrated that it is possible to induce *RAS*-mutant-specific T cell responses in both HDs and patients with *RAS*-mutant cancer by continuous antigen stimulations [18,23,24,25,27,33]. Interestingly, one study of *RAS* mutation-specific T cells in *RAS*-mutant patients failed to identify T cells specific to the patients’ corresponding mutations. However, several patients harbored T cells specific to mutant RAS epitopes that did not correspond to their own mutation. Additionally, in this study several *RAS* wt cancer patients had a high frequency of T cells specific to mutant RAS epitopes [15]. The authors suggest that the *RAS*-mutant-specific cells identified in patient cells were evidence of immuno-editing, and that transformed *RAS*-mutant cells had been cleared by specific T cells, whereas *RAS* wt cancer cells had not [15]. In another study, both CD4^+^ and CD8^+^ T cell lines specific to the G13D substitution were established, though the patient did not harbor this mutation; the authors explained this based on mutant cells harboring the G13D mutation being cleared by the immune system [33]. This process could also occur in HDs, as their T cells do not exert immuno-editing, but immune elimination. The evidence that these specific T cells have cleared transformed cells in HDs before the emergence of overt neoplastic disease is as follows: Firstly, T cells specific to mutant RAS epitopes have been shown several times to be able to recognize and kill both HLA-matched cancer cells with the corresponding *RAS* mutation and autologous or HLA-compatible cells that express the naturally processed mutant antigen [23,24,26,27]. As such, the HD T cells identified in our study are able to recognize cells that harbor the corresponding *RAS* mutation. Secondly, several vaccination trials directed at inducing an immune response specific to the *RAS* mutations have shown that vaccination with mutant RAS epitopes increases the number of specific T cells in the peripheral blood of patients [19,20,21,22,34]. Importantly, some trials have found that patients with a robust immune response to administered peptides have significantly improved overall survival [21,22,34]. In one study, *RAS*-mutant-specific immune responses were examined in long-term survivors from a trial in patients with pancreatic cancer who had been vaccinated with *RAS*-mutant peptide epitopes. Interestingly, all five long-term survivors presented an immune response to the vaccines, and three of the five patients had maintained their specific immune response more than seven years after inclusion in the study [30]. Taken together, these studies show that *RAS*-mutant cells can be killed by circulating *RAS*-mutant-specific T cells, and these T cells may have clinical significance because they clear transformed cells and induce remission.

The frequently occurring T cell responses we found in this study contradict other studies that failed to identify *RAS*-mutant-specific immune responses in HDs. There could be several explanations for this. Firstly, other studies have used several different protocols to investigate the occurrence of responses. In general, most of the other studies used ex vivo assays, in which cells are not stimulated in vitro before assaying. Notably, we identified some ex vivo responses. However, one should take into account that ex vivo responses in general are very difficult to detect even with highly sensitive methods, such as ELISPOT or tetramer analysis [35], Secondly, the peptides used in this study were long 19-mer epitopes that ensure coverage of a wide range of possible RAS-mutant epitopes. Given that antigens are processed and presented differently between individuals, we have ensured that a broad range of epitopes can be presented. Notably, RAS-mutant epitopes have been reported to bind several different HLA-II types [36]. Thirdly, we performed one in vitro stimulation, followed by stimulation with IL-2 the following day, and allowed the cells to incubate for 12 days before assaying. This allows sufficient time for specific T cells to expand and present a clear response, and the longer incubation time reduces the background activity mediated by the IL-2 stimulation.

The in vitro stimulation of T cells raises the question of whether the responses were induced responses or memory T cell responses. One needs to consider the fact that antigens processed and presented in PBMC cultures are not presented by highly functional antigen-presenting cells (APCs). The main APCs in PBMC cultures are monocytes and B cells, which are inferior to dendritic cells at presenting antigens, provide co-stimulation, and release pro-inflammatory cytokines in order to induce an immune response. One could speculate that the in vitro stimulation performed by us could potentially induce regulatory T cells, and not effector T cells, from naïve T cells, as suboptimal priming of naïve T cells generates regulatory T cells [37]. In addition, we chose to investigate the phenotype of the responding T cells by sorting CD4^+^ T memory cells into high-purity cultures. These memory T cells required co-culture with peptide-pulsed monocytes to demonstrate that antigen presentation by immature APCs is sufficient for a memory T cell response. The need for CD14^+^ monocytes in co-culture also suggests that RAS-mutant antigens require processing and presentation of cells with some APC potential. This is contrary to the neo-antigens derived from the *CALR* exon 9 mutations, as CD4^+^ memory cells do not need to be co-cultured with CD14^+^ monocytes to demonstrate a response [13]. In addition, ex vivo responses in HD PBMCs are more readily identified against mutant CALR neo-antigens than ex vivo responses against RAS-mutant epitopes. The rather infrequent ex vivo responses identified in this study could also be explained by this, as the 24 h and 48 h ex vivo assays may not allow sufficient time for monocytes and B cells to process and present RAS-mutant-derived epitopes to T cells.

Even though other studies have failed to identify HD responses to mutant RAS epitopes, the results in this study may not be that surprising, as several lines of evidence indicate that T cells in HDs recognize neo-antigens. The *NPM1* mutations identified in approximately 20% of patients with AML [38] generate a novel mutant NPM1 protein with a mutant C-terminus that is recognized by HD T cells, and stimulation of HD T cells with epitopes derived from the mutant C-terminus of NPM1 incites strong T cell responses [6]. However, the phenotype of the specific T cells has not been identified. Epidemiological data indicate that some HLA types may protect against the occurrence of certain cancers caused by specific driver mutations that generate immunogenic neo-antigens. Notably, HLA-A3 and HLA-B8 are underrepresented in *BCR-ABL*^+^ CML [3], and HLA-B*07, B*18, and B*40 protect against *NPM1*-mutant AML [4], Furthermore, HDs also have memory T cells specific to aberrantly phosphorylated antigens [39], and the newly identified HD T memory responses specific to *CALR* exon 9 mutations [13] add further impetus to the notion of the elimination of transformed cells in HDs by neo-antigen-specific T cells.

One obvious explanation to the high frequency of HD T cell responses specific to *RAS*-mutants reported in this study would be that the specific T cells cross-react with the wt epitope. Therefore, we included wt peptide-stimulated negative controls, and even though we found a decline in the frequency of some of the responses, several strong responses against mutant epitopes were still detected when wt peptides were used as negative controls. The establishment of T cell cultures that did not exhibit cross-reactivity with the wt epitopes showed that T cells specific to mutant RAS-epitopes harbor T cell receptors that recognize mutant but not wt RAS, and in one culture that showed cross-reactivity with the wt epitope, the cross-reactivity with the wt epitope disappeared when cells were stimulated with a lower concentration of peptide (Figure 3D). Hence, it would be interesting to test the avidity for some of the most immunogenic mutant peptides and compare with the avidity of the corresponding peptide. Two studies have identified the occurrence of T cells that cross-react with other mutant RAS epitopes and wt RAS without the occurrence of autoimmune phenomena [15,30]. Another explanation could be that some wt *RAS*-specific T cells escape thymic deletion [40], but the mechanisms of peripheral tolerance prevent autoimmune phenomena. Some of these T cells could cross-react with mutant RAS neo-antigens and explain that mutant *RAS* cells are deleted; however, as we demonstrated, some T cells do not exhibit any cross-reactivity with wt *RAS*. Thus, we conclude that these T cells have been challenged with mutant RAS neo-antigens. With our findings above at hand, it would be highly relevant to test immune responses in patients with *RAS*-mutant cancers and clarify if these patients display an attenuated immune response to the mutant epitopes, which would add impetus to the notion of the evolution of *RAS*-mutant cancer due to tumor immune evasion. The translational relevance of our findings would be to identify patients with premalignant *RAS*-mutant lesions, such as patients with precursor pancreatic cancer lesions [41], and perform vaccinations with *RAS*-mutant-derived epitopes to enhance the *RAS*-mutant-specific immune response, in order to facilitate clearing of the pre-malignant lesion.

## 4. Materials and Methods

### 4.1. Donors

Buffy coats from anonymized blood donors were acquired from the blood bank at Rigshospitalet, Copenhagen, Denmark. All participants provided informed consent before study entry, in agreement with the Helsinki Declaration. PBMCs were isolated with Lymphoprep (Axis Shield, Oslo, Norway) and frozen in fetal calf serum with 10% dimethyl sulfoxide (Sigma-Aldrich, St. Louis, MO, USA). According to the Danish Law on Ethics Committee §14, Section 3, the usage of anonymized biological material for research purposes does not need to be approved by an ethics committee [42].

### 4.2. Peptides

The six most common amino acid substitutions for the codon 12, 13, and 61 mutational hotspots were chosen. To expand the possibility of identifying an HLA-I restricted epitope (decamer or nonamer), we chose to work with 19-mer peptide epitopes with the amino acid substitution at the central position. For our initial screen of responses, we worked with crude peptides with a >70% purity. These were provided by Pepscan (Lelystadt, the Netherlands). For our subsequent detailed analysis of responses, including wt epitopes as negative controls, we used high-purity peptides with a >70% purity. These were provided by Schafer (Copenhagen, Denmark). All peptides were provided in lyophilized form and reconstituted in dimethyl sulfoxide (Sigma-Aldrich) to a stock concentration of 10 mm before use.

### 4.3. Enzyme-Linked Immunospot Assay

We used enzyme-linked immunospot (ELISPOT) assays on cells stimulated in vitro to identify immune responses against mutant RAS epitopes. In short, PBMCs were thawed and stimulated with mutant RAS epitopes. Cells were stimulated with 2 µL of 10 mM peptide in culture in 500 µL X-VIVO (Lonza, Belgium) for 2 h at 37 °C in a humidified 5% CO_2_ atmosphere. After incubation, 1.5 mL of X-VIVO with 5% human serum was added to the cell cultures and incubated as described above. The next day, IL-2 (Novartis, Switzerland) was added to the cells for a concentration in culture of 120 U/mL. The cells were then allowed to incubate for 12–14 days before being counted, set up in ELISPOT at the desired concentration, and stimulated with peptide for a final concentration of 5 µM in the well. The plates used for ELISPOT were polyvinylidene fluoride (PVDF) membrane plates (Merck, Germany) coated with primary IFN-γ specific antibodies (Mabtech, Sweden) the day before use. The next day, the wells were washed six times in 200 µL sterile PBS and the wells conditioned with X-VIVO. Next, the X-VIVO was poured out and effector cells added at 2–3 × 10^5^ cells/well in X-VIVO and stimulated with peptide as described above. The plates were incubated overnight. The next day, the cells were poured off, the wells washed six times with 200 µL PBS, and the wells coated with secondary antibody, streptavidin enzyme conjugate, and enzyme substrate, according to the manufacturer’s protocol (Mabtech, Sweden). The ELISPOT plates were allowed to dry overnight, and then analyzed and counted using the ImmunoSpot S6 Ultimate Analyzer (CTL Analyzers, Shaker Heights, OH, USA). The number of specific cells was defined as the mean number of spots in peptide-stimulated wells subtracted by the mean number of spots in negative control wells. All ELISPOTs were performed in triplicate, with three stimulated wells and three negative control wells. Statistical analysis was performed using the distribution-free resampling (DFR) method and the more conservative DFR(2x) method described by Moodie et al. [28]. The DFR method is recommended for the statistical analysis of ELISPOTs, and has a false-positivity rate of 5%, whereas it is only 1% for the more conservative DFR(2x) method [28]. Ex vivo ELISPOT assays were performed by resting thawed cells overnight and then plating the cells directly without any prior in vitro stimulation. Cells for ex vivo ELISPOT assays incubated for 24 or 48 h, in order to ensure an appropriate amount of time for antigen processing. Ex vivo ELISPOTs were performed either in triplicate or quadruplicate. In a few ex vivo experiments, we used tumor TNF-α ELISPOTs (Mabtech, Sweden), using the same methods as for the IFN-γ ELISPOT. Unless otherwise stated, the peptide concentration in the assays was 5 µM.

### 4.4. Intracellular Cytokine Staining

Primary responses were analyzed following the in vitro stimulation protocol used for ELISPOT. Upon assaying, cells were plated at a concentration of 5 × 10^5^/200 µL in 96-well, round-bottom plates, and then stimulated with peptide for a final concentration of 5 µM, before incubation at 37 °C with 5% CO_2_ in a humidified atmosphere. After 1 h of stimulation, Brefeldin A (BD Biosciences, San Jose, CA, USA) was added at a dilution of 1:1000. After another 4 h of incubation, cells were washed three times, and then stained with surface markers: anti-CD3-APC-anti-H7, anti-CD4-PerCP or anti-CD4-FITC, anti-CD8-FITC or anti-CD8-PerCP, anti-IFN-γ-APC, and anti-TNF-α-BV421 (all BD Biosciences, San José, CA, USA). Dead cells were stained with the Fixable Viability Stain 510 (BD Biosciences, San José, CA, USA). After 30 min of staining, cells were washed twice and permeabilized using a Fixation Permeabilization Kit (Thermo-Fisher, Waltham, MA, USA). Cells were permeabilized for at least 30 min, then washed twice and stained for IFN-γ and TNF-α, using the above antibodies diluted in a fixation permeabilization buffer. After 30 min of staining, cells were washed twice, and then acquired using a FACS CANTO flow cytometer (BD Biosciences, San Jose, CA, USA) with appropriate application settings and compensation controls. Unless otherwise stated, the peptide concentration in the assay was 5 µM.

### 4.5. Establishment of T Cell Cultures Specific to Mutant RAS Epitopes

Donor PBMCs were stimulated in vitro with IL-2 as described above. After 12–14 days of incubation, the cells were washed and plated in round-bottom wells in X-VIVO at a concentration of 5 × 10^5^ cells/200 µL. The cells in the culture were stimulated with peptide at a concentration of 5 µM and allowed to incubate overnight. The next day, the cells were washed and enriched using the CD137 enrichment kit (Miltenyi Biotech, Germany) according to the manufacturer’s protocol. The enriched cells were counted and cloned by limiting dilution in 100 µL REP mix in 200 µL round-bottom wells. The REP mix consisted of irradiated HD PMBCs from three different donors with a concentration of 10^6^ feeder cells/mL X-VIVO with 5% human serum. The REP mix also contained 0.03 µg/mL anti-CD3 (OKT3) and 6000 U/mL IL-2, allowing for at rapid expansion of the cloned cells. When the cultures started to expand, they were analyzed for a response to their cognate antigen and expanded for further analysis using a new REP. The G12A-specific culture established from donor BM-60 was established by stimulating donor PBMCs with G12A peptide directly, without any prior in vitro stimulation. The enriched cells were expanded using REP, and thus were not cloned.

### 4.6. CD4^+^CD45RO^+^ Memory T Cell Cultures

The enrichment and purity of enriched cultures were analyzed as described in a previous paper [13]. However, in this study, we needed to enrich for CD14^+^ monocytes and pulse with peptide to ensure proper antigen processing and presentation to the enriched memory cells. In short, HD PBMCs were thawed and enriched for CD14^+^ monocytes using the CD14^+^ enrichment kit (Miltenyi Biotech, Germany), according to the manufacturer’s protocol. The monocytes were then pulsed with peptide for 2 h, as described for ELISPOT, and the CD4^+^CD45RO^+^ memory T cells enriched from the monocyte-depleted cell fraction using the CD4^+^ memory enrichment kit (Miltenyi Biotech, Germany), according to the manufacturer’s protocol. The enriched cells were then re-suspended in 1.5 mL X-VIVO and added to the peptide-pulsed monocytes. A fraction of the culture was analyzed for enriched memory T cells using the antibody panel described previously [13]. After 12 days of in vitro culture, the cells were analyzed for immune responses against mutant RAS using IFN-γ ELISPOT and ICS, as described above.

## 5. Conclusions

HDs harbor T cells specific to epitopes derived from the tumor specific *RAS* mutations. T cells specific to mutant RAS are memory T cells suggesting that HD may spontaneously clear transformed cells harboring a *RAS* mutation and subsequently generate T-cell memory to the mutations.

## Figures and Tables

**Table 1:** Amino acid sequences of the peptides derived from mutant RAS used in this study. For the mutant epitopes, the substituted amino acid has been marked with a red letter.

CODON 12 PEPTIDES	Amino Acid Sequence
G12A:	EYKLVVVGAAGVGKSALTI
G12C:	EYKLVVVGACGVGKSALTI
G12D:	EYKLVVVGADGVGKSALTI
G12R:	EYKLVVVGARGVGKSALTI
G12S:	EYKLVVVGASGVGKSALTI
G12V:	EYKLVVVGAVGVGKSALTI
G12wt:	EYKLVVVGAGGVGKSALTI
**CODON 13 PEPTIDES**	
G13A:	YKLVVVGAGAVGKSALTIQ
G13C:	YKLVVVGAGCVGKSALTIQ
G13D:	YKLVVVGAGDVGKSALTIQ
G13R:	YKLVVVGAGRVGKSALTIQ
G13S:	YKLVVVGAGSVGKSALTIQ
G13V:	YKLVVVGAGVVGKSALTIQ
G13wt:	YKLVVVGAGGVGKSALTIQ
**CODON 61 PEPTIDES**	
Q61E:	LLDILDTAGEEEYSAMRDQ
Q61H:	LLDILDTAGHEEYSAMRDQ
Q61K:	LLDILDTAGKEEYSAMRDQ
Q61L:	LLDILDTAGLEEYSAMRDQ
Q61P:	LLDILDTAGPEEYSAMRDQ
Q61R:	LLDILDTAGREEYSAMRDQ

## Supplementary Materials

The following are available online at https://www.mdpi.com/2072-6694/12/10/3045/s1, Figure S1: Depiction of response from each of the ten healthy donors against the six different crude exon 12, 13, and 61 mutant epitopes, Figure S2: Depiction of response from each of the donors analyzed for response against the high purity G12A, G12C, G12D, G12R, G12S, G12V, G13R, and G13V-derived epitopes, where the negative control was the corresponding wt peptide, Figure S3: To clarify if the introduction of the wt epitope as a negative control decreased the amplitude of responses in peripheral blood mononuclear cells, we analyzed peripheral blood mononuclear cells from 18 different donors, in which both unstimulated cells (no wt control) and wt epitope (with wt control) were used as negative controls. Comparison of the response amplitudes between the two groups was performed using a paired *t*-test, Figure S4: FACS plots of CD8^+^ T cells in the PBMC cultures with a CD4+ T cell response to mutant RAS epitopes displayed in 1D, Figure S5: Gating strategy used to assess the purity of the enriched memory cell fractions with the figure, showing the gates set for the CD45RO stained cells and the CD45RO fluorescence minus one (FMO) control, respectively. Figure S6: (A) Purity analysis of the enriched CD4^+^CD45RO^+^ memory T cells mixed with peptide-pulsed autologous CD14^+^ monocytes. The gate is set for live CD3^+^CD4^+^ cells to investigate the purity of the enriched cells. (B) FACS plots from an intracellular cytokine stain, in which enriched memory cells were stimulated with mutant epitope (top) or wt epitope (bottom). (C) IFN-γ ELISPOT assays, with a graphical depiction of the response (top) and representative wells of the response (bottom).
