# Supplementary material for: Healthy Donors Harbor Memory T Cell Responses to RAS Neo-Antigens
Source: Cancers (Basel). 2020 Oct 19;12(10):3045. doi: 10.3390/cancers12103045 (PMC7589254; doi:10.3390/cancers12103045)
Supplement: Supplementary file 1 [file cancers-12-03045-s001.pdf]

## Supplementary Materials

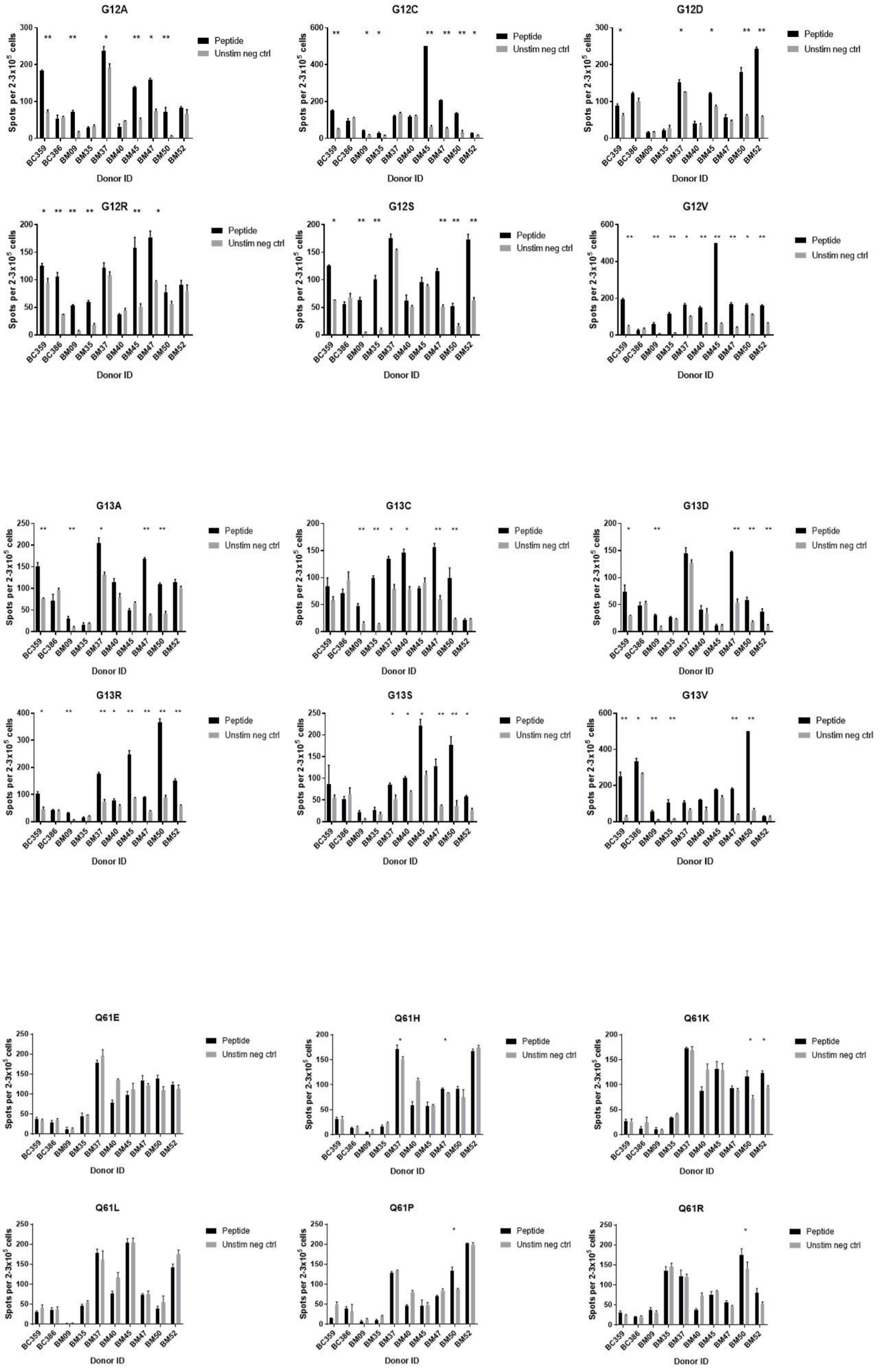


**Figure S1.** Depiction of response from each of the ten healthy donors against the six different crude exon 12, 13, and 61 mutant epitopes.

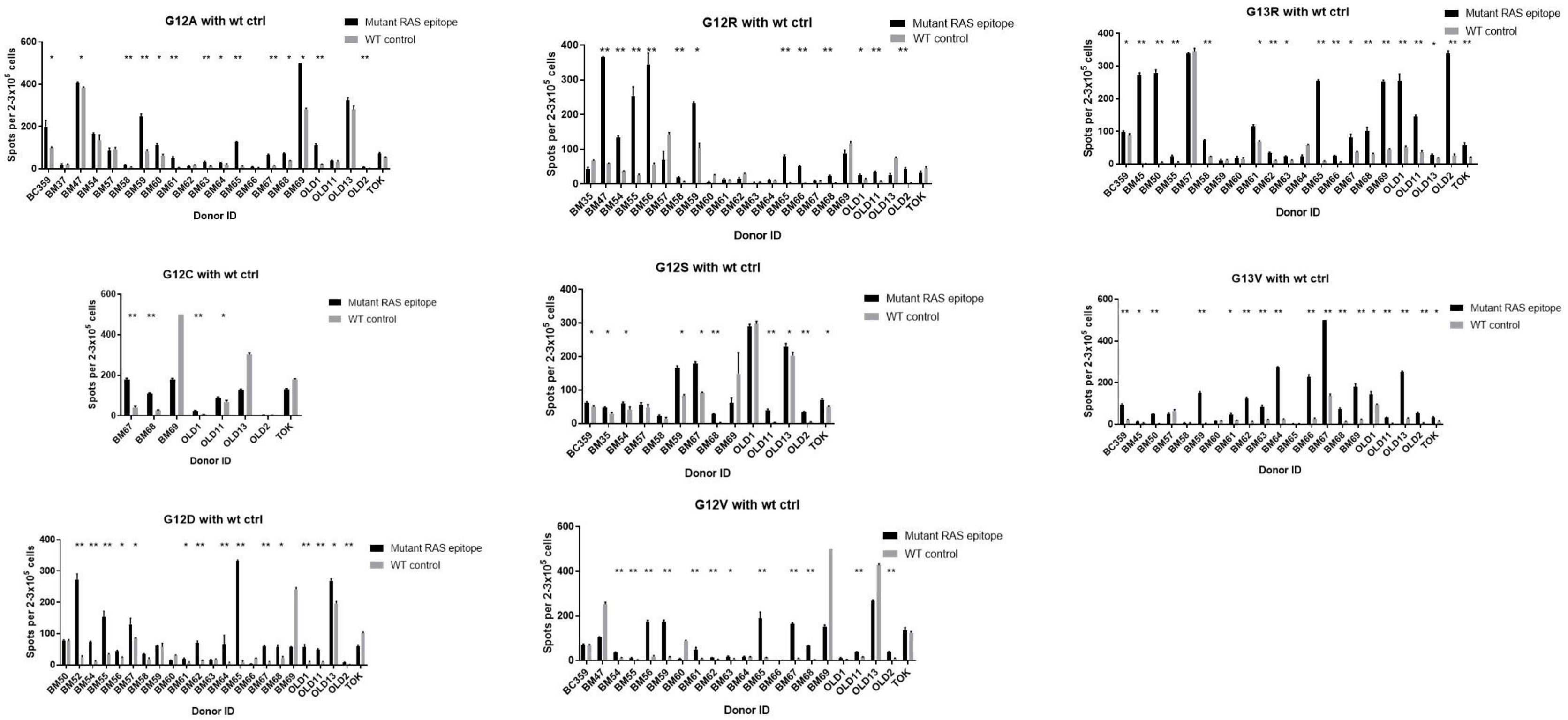


**Figure S2.** Depiction of response from each of the donors analyzed for response against the high purity G12A, G12C, G12D, G12R, G12S, G12V, G13R, and G13V-derived epitopes, where the negative control was the corresponding wt peptide.

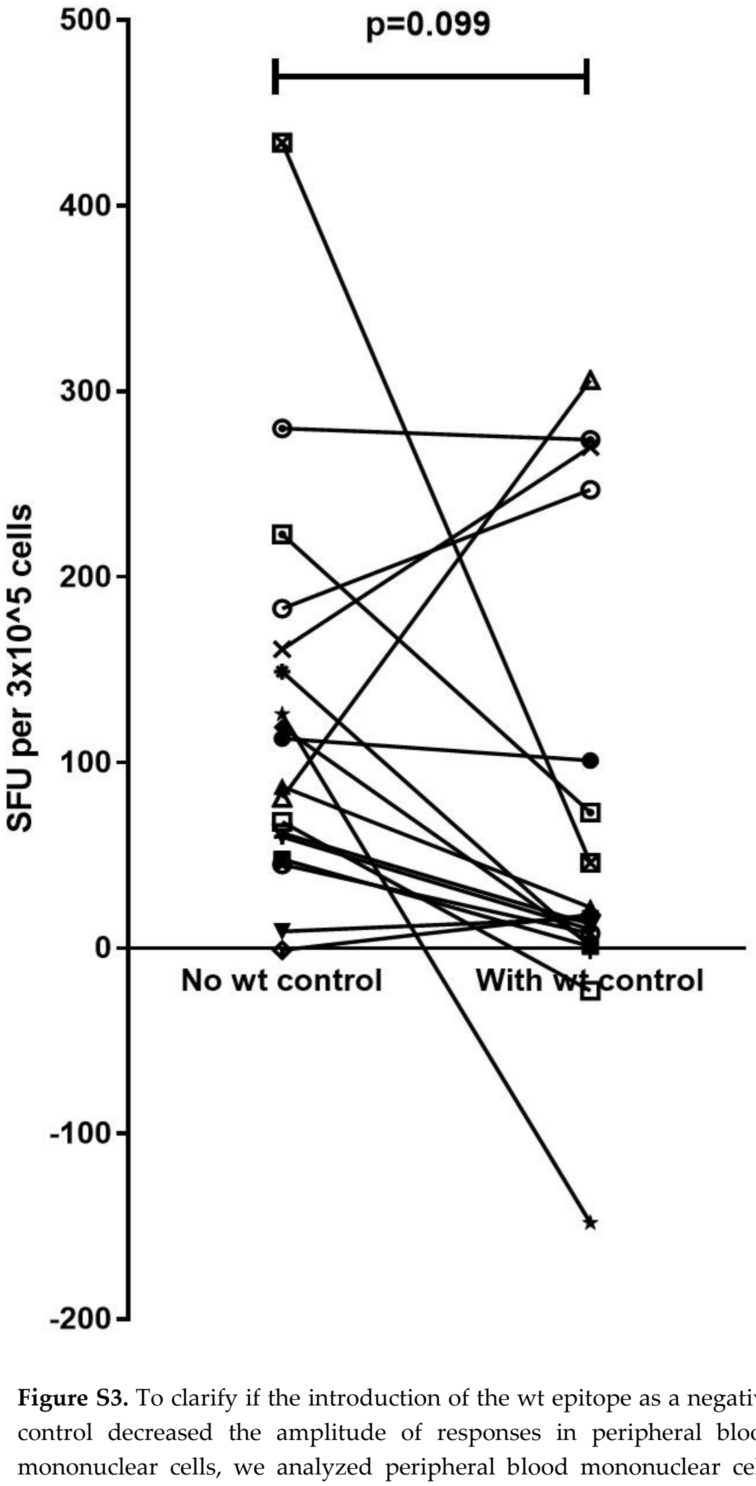


**Figure S3.** To clarify if the introduction of the wt epitope as a negative control decreased the amplitude of responses in peripheral blood mononuclear cells, we analyzed peripheral blood mononuclear cells from 18 different donors, in which both unstimulated cells (no wt control) and wt epitope (with wt control) were used as negative controls. Comparison of the response amplitudes between the two groups was performed using a paired *t*-test.

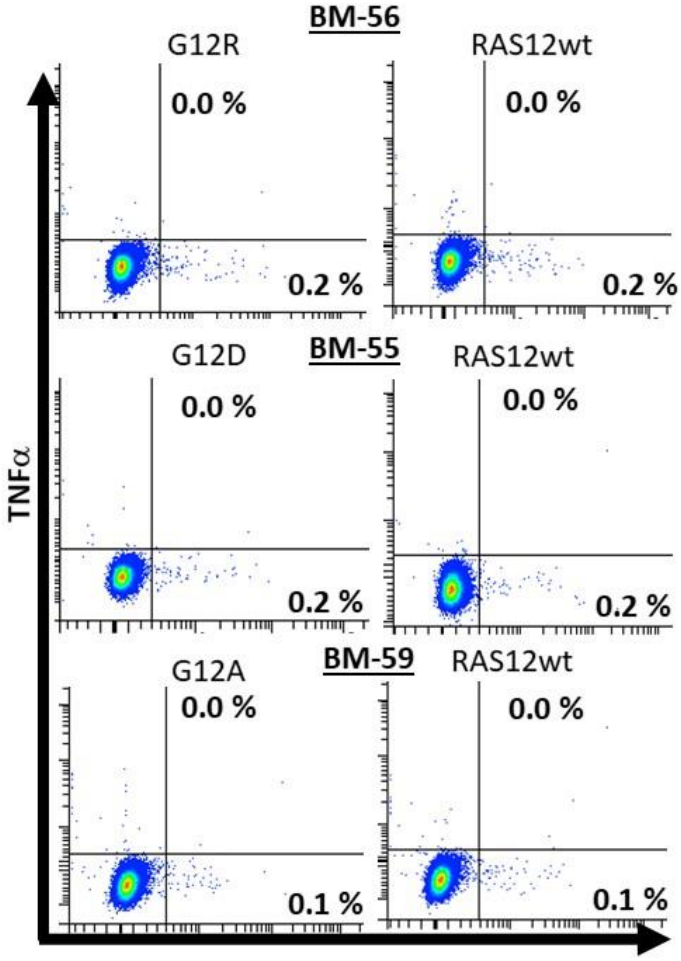


**Figure S4.** FACS plots of CD8⁺ T cells in the PBMC cultures with a CD4+ T cell response to mutant RAS epitopes displayed in 1D.

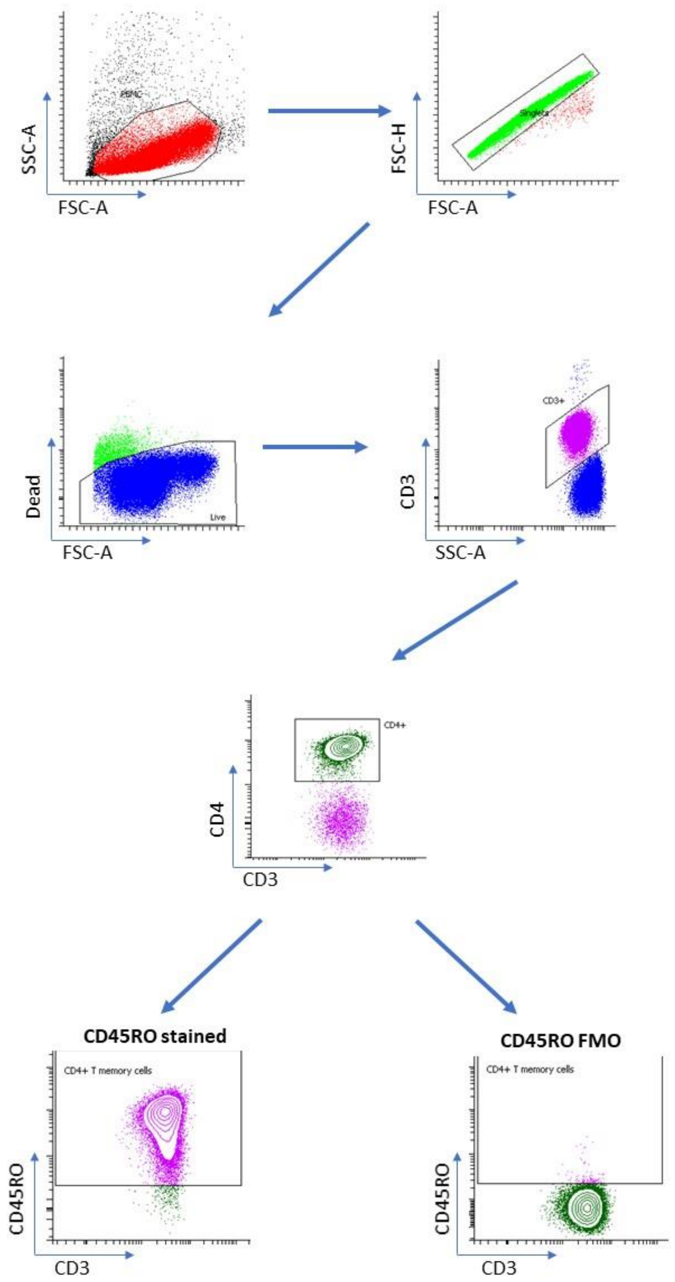


**Figure S5.** Gating strategy used to assess the purity of the enriched memory cell fractions with the figure, showing the gates set for the CD45RO stained cells and the CD45RO FMO control, respectively.

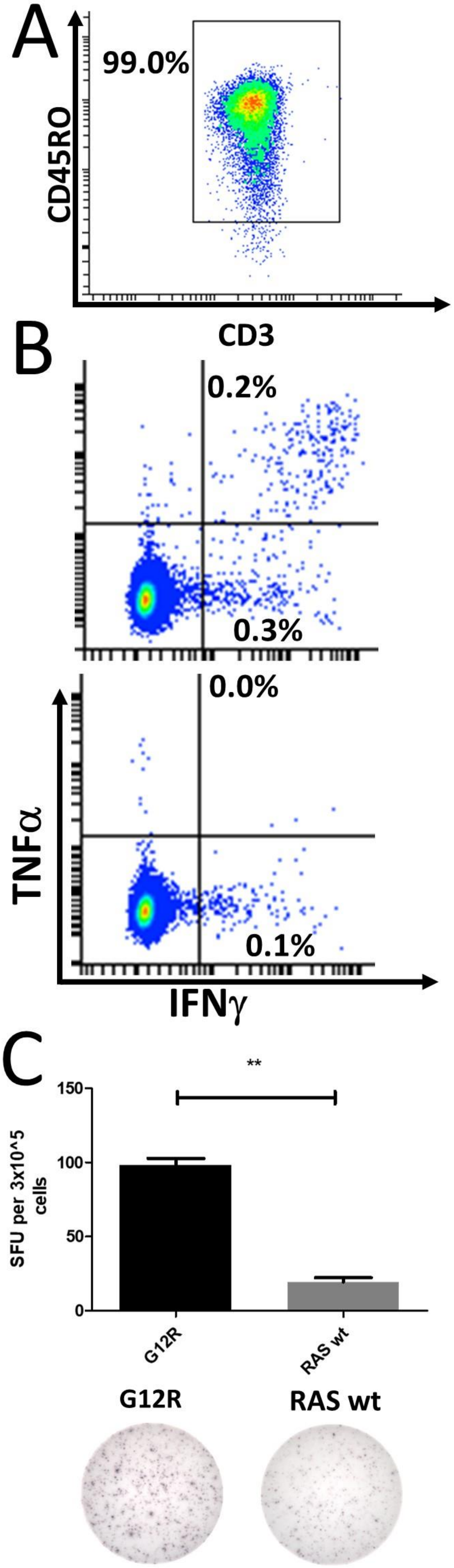
A
99.0%
CD45RO
CD3
B
0.2%
0.3%
0.0%
0.1%
TNFα
IFNγ
C
**
150
100
50
0
SFU per 3x10^5 cells
G12R
RAS wt
G12R
RAS wt

**Figure S6:** (**A**) Purity analysis of the enriched $CD4^+CD45RO^+$ memory T cells mixed with peptide-pulsed autologous $CD14^+$ monocytes. The gate is set for live $CD3^+CD4^+$ cells to investigate the purity of the enriched cells. (**B**) FACS plots from an intracellular cytokine stain, in which enriched memory cells were stimulated with mutant epitope (top) or wt epitope (bottom). (**C**) IFN-γ ELISPOT assays, with a graphical depiction of the response (top) and representative wells of the response (bottom).
